# Biochemical Activation and Regulatory Functions of Trans-Regulatory KLF14 and Its Association with Genetic Polymorphisms

**DOI:** 10.3390/metabo13020199

**Published:** 2023-01-29

**Authors:** Muhammad Sajid Hamid Akash, Sumbal Rasheed, Kanwal Rehman, Muhammad Ibrahim, Muhammad Imran, Mohammed A. Assiri

**Affiliations:** 1 Department of Pharmaceutical Chemistry, Government College University, Faisalabad 38000, Pakistan; 2 Department of Pharmacy, The Women University, Multan 60000, Pakistan; 3 Department of Applied Chemistry, Government College University, Faisalabad 38000, Pakistan; 4 Research Center for Advanced Materials Science (RCAMS), King Khalid University, Abha 62413, Saudi Arabia; 5 Department of Chemistry, Faculty of Science, King Khalid University, Abha 62413, Saudi Arabia

**Keywords:** KLF14 gene, single nucleotide polymorphism, genetic variations, ApoA1, KLF14 gene polymorphism

## Abstract

Krüpple-Like family of transcription factor-14 (KLF14) is a master trans-regulatory gene that has multiple biological regulatory functions and is involved in many pathological mechanisms. It controls the expressions of several other genes which are involved in multiple regulatory functions. KLF14 plays a significant role in lipid metabolism, glucose regulation and insulin sensitivity. Cell apoptosis, proliferation, and differentiation are regulated by the KLF14 gene, and up-regulation of KLF14 prevents cancer progression. KLF14 has been used as an epigenetic biomarker for the estimation of chronological age due to the presence of different age-related CpG sites on genes that become methylated with age. Different genome-wide association studies have identified several KLF14 variants in adipose tissues. These single nucleotide polymorphisms in KLF14 have been associated with dyslipidemia, insulin resistance, and glucose intolerance. Moreover, the prevalence of genetic polymorphism is different in different populations due to ethnic differences and epigenetic modifications. In addition, environmental and physiological factors such as diet, age, gender, and obesity are also responsible for genetic mutations in KLF14.

## 1. Introduction

KLF14 is a member of the Krüpple-Like family of transcription factors [1,2], which regulates normal gene expression in mammals. In total, there are eighteen KLF proteins that are further grouped into three families. KLF14 activates transcription but also possesses a repressive role by interacting with Sin3A, which is a co-repressive protein in DNA [1]. KLFs have different patterns of expression in different body tissues by which many exhibit pronounced expressions, including KLF6 [3], KLF7 [4], KLF9 [5], KLF10 [6], KLF11 [7], KLF13 [8], and KLF15 [9]. While some members have tissue-specific expression, e.g., KLF1 expresses in megakaryocytes and red blood cells, KLF2 expresses in white adipose tissues, and KLF4 and KLF5 express in blood vessels and white adipose tissues [10].

KLF14 is a master trans-regulatory gene linked with the expression of various multiple metabolic traits. Mutations in master regulatory genes are often linked with the incidence of severe diseases as these genes regulate the concurrent expression of several genes [11]. Several kinds of metabolic disorders, including diabetes mellitus (DM), cardiovascular diseases (CVDs), and especially coronary artery diseases (CADs), are strongly linked with the variants near the KLF14 gene which is located on chromosome 7. KLF14 variants have more frequent expressions in women than in men. It has been evidenced from several studies that environmental factors directly influence the normal expression of the KLF14 gene and play a significant role in altered metabolic processes [10].

KLF proteins regulate several cell signaling pathways, including apoptosis, proliferation, and differentiation, etc. [1]. Only the maternal allele of KLF14 is expressed in humans and KLF14 expression has been found in many organs and tissues, including adipose tissues, the liver, the brain, and muscles [12]. KLF14 regulates insulin secretion, lipid metabolism, inflammatory responses, cell differentiation, and proliferation. Therefore, it is proven as a potential target to reduce the risk of CVDs [13]. Moreover, KLF14 regulates the efflux of HDL-C and ApoA-1; therefore, alterations in the normal expression of KLF14 triggered by gene polymorphism or epigenetic alteration induce the onset of metabolic disorders [14]. Several KLF14 variants have been identified using genome-wide association studies (GWASs), which lead toward the altered insulin sensitivity, development, and progression of several metabolic diseases, including DM, myocardial infarction, atherosclerotic cardiac diseases, and ischemic stroke [15,16,17,18]. Interestingly, single-nucleotide polymorphisms (SNPs) of KLF14 were significantly found in adipose tissues [15,19]. KLF14 has different CpG sites which can be hypermethylated over age, thus KLF14 can be used to estimate the chronological age and serve as an age-related epigenetic biomarker [20]. KLF14 is widely expressed in various tissues, and its role in different altered metabolic processes has been reviewed. We have previously worked on genetic polymorphism of different genes, including FTO, PPAR-γ, ABCC8, APOEε4, AGT, and MTHFR genes [21,22,23,24]. This article summarizes the genetic polymorphisms in KLF14 and the role of the KLF14 gene in transcriptional regulation, age prediction, progression of metabolic disorders, glucose and lipid metabolism, and cancer suppression.

## 2. Configuration and Expression of the KLF14 Gene

KLF proteins bind with the regulatory regions of a targeted gene as these proteins have conserved C_2_H_2_-type zinc finger domains at their C-terminal, which is involved in binding with the GC-rich regions of the genes [1]. The KLF14 protein retains three C_2_H_2_-type zinc finger structural motifs in C-terminus with two domains (first and second) and a short third domain encompassing 25 and 23 amino acid residues, receptively [25,26]. These three zinc finger domains can identify three DNA base pairs, and thus have an affinity to bind at three sites in the gene regulatory region [27]. Moreover, it has been found that these domains possess positively charged amino acids which may assist in the localization of the nucleus. Several investigational studies concluded that KLF proteins can interact with similar GC-rich sequences or 5′-CACCC-3′ of several genes’ promoter regions [28,29]. KLF proteins show binding affinity with other proteins’ associates such as co-repressors, histone-modifying enzymes, and co-activators, etc., due to their N-terminus regions [30].

The transcriptional target sites of KLF14 are not well described however, few studies have identified different targets. Liver-specific KLF14 knockout in mouse models causes ApoA-1 deficiency, which indicates that KLF14 is involved in the transcriptional regulation of ApoA-1 [31]. Therefore, ApoA-1 is an important target of KLF14. There are two binding sites for KLF14 on the promoter region of ApoA-1 in humans. Sphingosine kinase 1 (SK1) is also a functional transcriptional target of KLF14. KLF14 suppresses the transcription of SK1 by binding with its promoter region [32]. One study demonstrated that KLF14 at the FOXP3 Treg-specific demethylation region remolds the chromatin and, as a result, regulates T-cell differentiation [33].

Trans-regulated genes’ promoter regions have enriched binding motifs for KLF14 [10]. These trans-genes regulate the MetSyn such as IDE, a regulator of insulin degradation [34], SLC2A4, which regulates the expression of GLUT4 protein which is responsible for insulin-induced glucose uptake in muscles and adipose tissues [35], and STARD10 which regulates insulin secretion in β-cells of pancreatic islets [36,37] as shown in Table 1. Moreover, KLF14 regulates the MAPK proteins level as KLF14 knockout results in the elevated level of MAPK proteins, including p38 and ERK1/2, which are associated with the regulation of several pro-inflammatory factors [38].

## 3. KLF14 Trans-Regulatory Network

The KLF14 gene is located on chromosome 7q32.3. Out of 82 KLF14-linked transcriptional factors, only 62 have been identified in humans. Transcriptional factors are specialized proteins that control gene expression. These factors are encoded by different genes, including HOXA9, HOXA3, and AHR. The Sin2A protein represses transcription, which leads to the transcriptional repression of important enzymatic proteins such as HDAC1 and HDAC2, DNA binding proteins, such as Mas and MeCP2, and the co-repressor proteins Ikaros and SMRT [42,43].

The major family of transcriptional factors, including sequence-specific DNA proteins and genes, have an important role in the regulation of different body functions, including CNS development (EN1), hematopoiesis (EVI1), cell growth regulation (MEF2A), regeneration of muscle (MYOD1), cell apoptosis (EVI1, MEF2A), and erythroid development (GATA1), and are vital in normal growth (MEIS1), hematopoietic proliferation and development, and endocrine cell lineage (GATA2), and embryonic regulation (FOXC1), etc. Interestingly, important genes that are involved in the pathogenesis of metabolic disorders such as the TCF7L2 and PPARG genes are not included among these genes.

The trans-acting regulator, encoded by the KLF14 gene regulates the expression of a cluster of genes that are involved with the metabolic phenotypes, including HDL level, insulin and glucose levels, LDL level, TG level, BMI, and insulin sensitivity index, etc. [11]. The resultant product (transcriptional factor) of KLF14 gene transcription interacts with 10 genes and establishes the protein–protein interactions with 32 proteins, which have a strong association with metabolic disorders and several other biological functions [44]. These 32 proteins, including CKAL1, KCNQ1, IGF2BP2, etc., have shown significant interaction with the KLF14 protein. Studies have shown that the KLF14 protein interacts with UBC (polyubiquitin precursor) which regulates the cell cycle, endocytosis, apoptosis, cell signaling pathways, DNA repair, and protein trafficking, etc. [11].

## 4. Role of KLF14 in the Progression of Metabolic Disorders

KLF14 expression in adipose tissue has been associated with GWAS-associated SNPs and is related to an increase in disease risk. However, it requires more research and clarity to identify which kinds of cells are liable for KLF14 effects on disease risk. In subcutaneous adipose tissue, KLF14 is the main regulator of gene expression. The KLF14 gene undergoes genetic imprinting, and the only inherited allele from the mother is phenotypically expressed [45]. Therefore, only maternally inherited SNPs have substantial connotations with KLF14′s expression in adipose tissue. Although, in many tissues, the KLF14 gene imparts its expression but only in the adipose tissues, genetic variants regulate the KLF14 expression [10]. KLF14 transcription factor in GWAS locus is a casual gene, which has regulatory mechanisms in adipose tissues. Moreover, T2DM has been linked with the SNPs KLF14 gene expression in adipose tissues, and in the KLF14 gene locus near the transcription promoter site. The presence of an adipose-specific enhancer has been associated with T2DM (https://gnomad.broadinstitute.org/, accessed on 15 December 2022).

T2DM has a high prevalence around the globe and is characterized by several associated risk factors notably insulin resistance, glucolipotoxicity, and impaired insulin secretion, which leads to the malfunctioning of different body organs. KLF14 gene expression has been altered during the development and progression of metabolic diseases [38]. KLF14 plays important role in insulin sensitivity regulation. According to an investigational study, it has been found that KLF14 up-regulates insulin sensitivity in high-fat-diet-fed mice and obese mice via stimulation and activation of the PI3K/Akt signaling pathway [46]. KLF14 gene up-regulation via activation of fibroblast growth factor 2 leads to the inducement of SK1 expression [32]. SK1 regulates lipid catalyzation via the generation of lipid second messenger which ultimately induces insulin resistance [47]. Unnecessary fat deposition in the body results in the expansion of adipose tissues via a rise in the number and/or size of adipocytes [48]. However, to accommodate excess calorie intake, subcutaneous adipose tissues are unable to expand properly which results in fat deposition in the liver, skeletal muscles, and visceral fat drops which eventually induce insulin resistance; an ultimate lead towards metabolic disorders [49,50,51]. In subcutaneous adipocytes, KLF14 levels are higher in females than in males [10].

Several studies have found that epigenetic alterations in the KLF14 gene may induce metabolic disorders. An investigational study on mice models has evidenced that aging and obesity can down-regulate KLF14 expression. As in the adipose tissues of HFC-fed mice and aged wild-type mice, hypermethylation in the KLF14 promoter region was observed [52]. Similarly, another study revealed that KLF14 hypermethylation has been strongly associated with aging, insulin secretion, and the emergence of T2DM [53]. At the KLF14 gene, risk alleles of T2DM have a strong association with TGs and HDL-C levels. However, in the regulation of cholesterol metabolism, the role of the KLF14 gene is not well-elaborated. However, studies have found that due to the presence of risk alleles at KLF14, the HDL-C level has significantly decreased which increases the risk of DM [10,54]. It has been revealed from an experimental study that the low HDL-C level is a sex- and genetic-specific trait and the KLF14 gene is more specific to low HDL-C for females than males [10].

## 5. Role of KLF14 in Glucose Regulation

**Figure 1:**
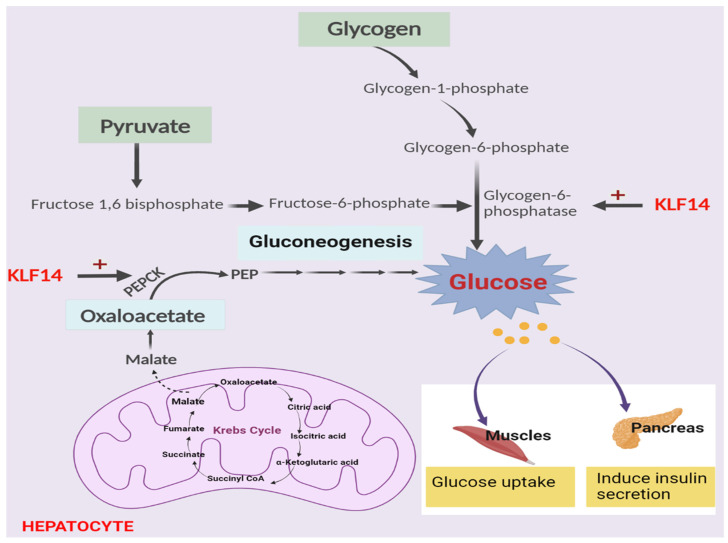
Regulation of gluconeogenesis by KLF14 transcriptional factor.

The liver regulates the glycolysis and gluconeogenesis processes via the secretion of insulin and glucagon hormones. Several studies have suggested that hepatic KLF14 regulates gluconeogenesis as up-regulation of KLF14 gene expression facilitates glucose production (Figure 1). KLF14 modulates hepatic glucose metabolism via the regulation of PGC-1α activity. A study has been conducted on mice models and primary hepatocytes to investigate the association between KLF14 gene expression and hepatic gluconeogenesis, insulin sensitivity, and glucose tolerance. In primary hepatocytes, KLF14 over-expression induces gluconeogenesis via up-regulation of peroxisome proliferator-activated receptor-γ coactivator 1α (PGC-1α) and mRNA levels. Contrariwise, KLF14 knockdown in hepatocytes results in reduced PGC-1α and glucose levels. In the livers of diabetic mice, KLF14 knockdown improved glucose tolerance [55].

Over-expression of KLF14 induces protective effects against the inhibition of glucose uptake stimulated by hyperglycemia and hyperinsulinemia in diabetic patients. The KLF14 gene is downregulated in diabetic humans and mice. Whereas KLF14 over-expression in hepatic cells induces glucose uptake mediated by insulin via activation of the PI3K/Akt signaling pathway. Moreover, the elevated level of glucose in hepatocytes is due to the direct binding and activation of KLF14 with the PGC-1α promoter. PGC-1α is a transcriptional factor involved in the gene expression of several different important gluconeogenic enzyme genes such as PEPCK encoding gene Pk1 and G6Pase encoding gene G6PC gene. So, the KLF14 gene is suggested as a strong positive glucose regulator in the liver [56].

## 6. Role of KLF14 in Lipid Metabolism

GWAS has demonstrated that genomic alterations near the KLF14 gene locus have a significant association with altered HDL-C levels, metabolic disorders, and heart ailments more specifically CADs. However, the exact mechanism of the KLF14 gene regulation of lipid metabolism and its associated impacts on atherosclerosis is not well elaborated. In an investigational study, both KLF14 over-expression and down-regulation in a dyslipidemia mouse model have been studied, which concluded that hepatic-KLF14 gene deletion leads to decrease serum HDL-C levels. While the cholesterol and HDL-C efflux capacity is increased by KLF14 modulated up-regulation of ApoA-I expression in perhexiline (KLF14 activator) treated WT mice. Perhexiline regulates the KLF14 pathway, and the results revealed that this intervention diminishes the development of atherosclerotic lesions in the apolipoprotein E-deficient mice which have a high cholesterol level and more chances for the development of atherosclerosis. The apolipoprotein E-deficient mice have a high cholesterol level and high chances for the development of atherosclerosis, whereas perhexiline decreases systemic cholesterol levels via activation of the KLF14 pathway [31].

Lipids are transported in the bloodstream in the form of lipoproteins, including HDL and LDL. Interestingly, cholesterol is mostly transported via HDL and LDL in animals and humans, respectively [57,58]. A meta-analysis has revealed that risk alleles at the KLF14 gene are associated with reducing the HLD-C level, KLF14 expression in adipose tissues, and increased T2DM risk, which suggested that an increased level of KLF14 has a protective effect against the risk of development and progression of metabolic disorders [10].

KLF14 has protective effects against inflammatory stress. According to an investigational study, it has been found that in human endothelial cells, KLF14 over-expression suppresses inflammation via modulation of TNF-α and IL-1β. KLF14 constrains the activation of endothelial cells via downregulation of the p65 subunit in the NF-κB signaling pathway. Thus, it has been concluded that in the endothelial cells, KLF14 restricts the macrophage-mediated inflammatory response by down-regulating the NF-κB signaling pathway [59].

## 7. KLF14 Role in Reverse Cholesterol Transport

**Figure 2:**
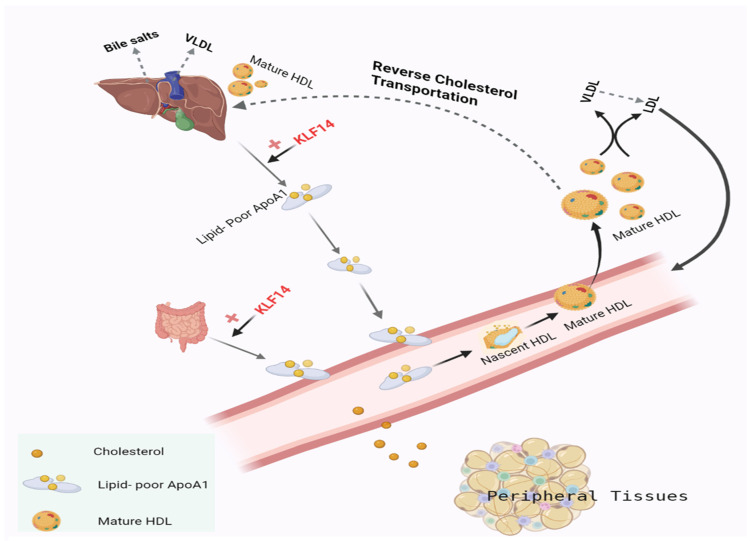
Role of KLF14 in the regulation of reverse cholesterol transport.

KLF14 is a direct ApoA-1 transcriptional regulator, which has been associated with the regulation of serum HDL levels and cholesterol transport [56]. In the liver, KLF14 over-expression elevates the serum HDL-C via the inducement of ApoA-1 [31]. Likewise, hepatic KLF14 knockdown has reduced the serum HDL-C level. There is an inverse relationship between the plasma HLD-C level and CADs [60]. ApoA-1 is responsible for the transport of cholesterol in the form of HDL from peripheral tissues (i.e., muscles) to the liver [61], as shown in Figure 2. This reverse cholesterol transport is considered as the main mechanism involved in HDL-C-associated anti-atherogenic effects. Thus, the up-regulation of hepatic KLF14 expression imparts protective effects against atherosclerosis [62]. Perhexiline is an activator of KLF14 expression, used as a prophylactic anti-anginal agent, which increases HDL-C levels and represses the risk of atherosclerosis [31].

The ability of peripheral body organs, especially adipose tissues and muscles, to uptake glucose is called peripheral insulin sensitivity, which is significantly affected by KLF14 [63]. KLF14 increases insulin sensitivity in the liver and KLF14 over-expression in Hpa1-6 cells up-regulates insulin-stimulated glucose uptake and Akt phosphorylation. Moreover, in the skeletal muscles, KLF14 increases GLUT4 translocation and insulin sensitivity [43].

## 8. Role of KLF14 in Insulin Sensitivity

The KLF14 gene belongs to the KLF family of zinc-finger binding proteins which regulates the normal biological functions of the body, including growth, proliferation, and differentiation. According to an in vivo investigation on mice, it has been found that decreased expression of KLF14 expression up-regulates the pre-adipocyte proliferation and interrupts normal lipogenesis, which results in the progression of T2DM, dyslipidemia, and insulin resistance. Furthermore, KLF14 risk alleles lead to body fat transfer into the abdominal stores from the gynoid that causes a prominent increase in the sizes of adipocytes, which ultimately disrupts the fat distribution and induces T2DM [15].

Numerous studies have demonstrated that in non-diabetic individuals, the risk alleles of KLF14 genes for T2DM are associated with insulin resistance characterized by increased fasting insulin. Moreover, a decreased level of HDL-C is linked with KLF14 gene risk alleles and leads to the development of CVDs [17,54]. In adipose tissue, KLF14 gene expression has been associated with combined insulin resistance characterized by elevated levels of TGs and fasting insulin and decreased level of HDL-C [64]. It has been concluded, using large data collected from GWAS and meta-analytical studies about metabolic traits, that KLF14 has strong association with phenotypes of metabolic syndromes, including insulin resistance, LDL-C, TGs, HOMO-IR, and the waist-hip ratio [65].

## 9. Role of KLF14 in Human Age Prediction

Epigenetic modifications in the KLF14 gene have been associated with age estimation in humans. DNA methylation is a useful and important epigenetic marker. The studies revealed that occurrence of DNA methylation has been associated with the restriction of gene expression [66,67]. Numerous environmental and endogenous factors stimulate the epigenetic modifications, including smoking, diet, stress, ethnicity, and nutrition. In the KLF14 gene, several age-related CpG regions have been identified in humans, and in an investigational study, such CpG sites were examined from blood and saliva samples. CpG sites were determined by developing age-predictor models using multivariate linear regression analysis. Usually, in humans with aging, DNA methylation decreases, while some genes have DNA methylation inducement with aging [20,68].

KLF14 is useful for pyrosequencing-based age estimation. KLF14 is a master trans-regulator gene; however, the gene sites which are involved in the determination of age are not actively involved in the transcriptional events. Gradual induction and loss of DNA methylation occur in response to slow stochastic drift [20]. Previously, several Cpg sites in these loci, including cg06493994 and cg04528819, have been determined. The age-related DNA methylation modifications have been examined in saliva, blood, brain, and other body tissues. An easy analytical technique to determine DNA methylation is quantitative pyrosequencing, which measures the relative methylation at high accuracy by using a minimum sample quantity [69].

In the determination of DNA methylation, major problems have arisen as a result of reproducibility against various kinds of cells. Moreover, it has been reported that modifications in DNA methylation are tissue-specific [70,71]. The identification of a universal age predictor marker for all cell types is difficult to find; however, one of the stable age predictors for several cell kinds is the ELOVL2 [72,73,74]. So, further investigation is required in this field to test the cross-correlation between different kinds of cells to determine more specific CpG sites set for age prediction.

## 10. Role of KLF14 in Cancer Suppression

Several studies have found that elevated KLF14 levels mitigate cancer progression and loss of the KLF14 gene triggers tumorigenesis. A study on KLF14’s role in the suppression of colorectal cancer revealed that increased expression of KLF14 induces apoptosis in cancerous cells. KLF14 repressed the growth of colorectal cancer by interacting with miR-374a-3p. Moreover, these findings suggest that the circTADA2A/miR-374a-3p/KLF14 axis may be used as a prognosis marker and the target for the treatment of colorectal cancer [41]. Another study has found that KLF14 suppressed the progression of colorectal cancer by targeting the HAND2-AS1/miR-1275 axis. HAND2-AS1 is adjacent to HAND1 which is lnsRNA transcribed antisense. HAND2-AS1, present on chromosome 4q, has onco-suppressive potential and is negatively associated with metastasis [75].

In cancerous cells, centrosome amplification has been frequently detected and is known as a hallmark of cancer. Polo-like kinase 4 (PLK4) is a key modulator for duplication and the assembly of the centriole. Over-expression of PLK4 results in centrosome amplification, while the KLF14 gene repressed the transcription of PLK4, and studies found that the loss of the KLF14 gene results in PLK4-induced centrosome amplification. Several studies have reported that in different kinds of human neoplasms, an elevated PLK4 transcription and decreased KLF14 expression were found [76]. KLF14 over-expression leads to mitotic catastrophe and induces both necrosis and apoptosis in cancerous cells. Over-expression of KLF14 induces cellular demises and insufficient cell cycle checkpoints, particularly spindle assembly and DNA structure checkpoints [77]. Normally, KLF14 gene expression is downregulated in cancer, and thus the induced KLF14 expression leads to mitotic arrest. KLF14 down-regulation induces genomic instability, while the up-regulation of KLF14 results in mitotic catastrophe. These findings suggest that KLF14 can be used as a prognostic biomarker and a target for the diagnosis and treatment of cancer [76].

## 11. Single Nucleotide Polymorphisms of KLF14

SNPs may have different associations with a disease in different populations due to ethnic differences or geographic disparities which induce variations in genomic material. The inconsistent outcomes may vary due to environmental factors, diet structures, and lifestyle differences. Even in the same province of China, different populations show contrary associations with disease. In 2016, a study was conducted to determine the association of T2DM with KLF14 gene polymorphism along with three different genes in the Han Chinese population in China. In this study, 736 diabetic patients and 768 controls were recruited, and SNP genotyping was performed using TaqMan SNP genotyping fluorescence quantitative assays. A chi-test was performed for statistical analysis. Their findings suggest that KLF14 rs972283 SNP has not been associated with T2DM in the Han Chinese population in Henan province, China. Therefore, it has been concluded that the KLF14 rs972283 variant is not a risk factor for T2DM in the Han Chinese population [78].

A meta-analysis has been performed in which data from the worldwide literature from 2008 to 2013 were collected and heterogeneity was performed. In this study, 5 articles with 106,535 controls and 50,552 cases were investigated for rs972283 in the KLF14 gene. For data analysis, the Metan module in STATA 11.0 was used while the SNP association with T2DM was determined using 95% CIs and ORs. Heterogeneity in the pre-conducted studies was calculated using the heterogeneity index (I2, 0–100) where I2 > 50%, considering a high level of heterogeneity. The Egger regression approach and funnel plots were used to investigate publication bias. The results showed that the gene KLF14 risk allele G of rs972283 was strongly associated with a significant susceptibility to T2DM globally [79].

Similarly, another case-control study has been conducted in the Iranian population with 512 controls and 475 cases to determine the association between KLF14 rs76603546 and the progression and risk of T2DM. Moreover, KLF14 rs76603546 association with body weight, cholesterol, HOMO-IR, and Hb1Ac was analyzed using the one-way ANOVA method. For the genotyping study, RFLP-PCR was used. SPSS software was used for data analysis, and the chi-square test was used to test the SNP genotype frequencies. A logistic regression model was used to analyze the association between genotypes and T2DM, adjusted for body height, BMI, Hb1Ac, and body weight. This investigational study reported the KLF14 rs76603546 association with Hb1Ac and T2DM in the targeted population [80].

GWAS on the Pakistani population has revealed that 13 variants increase the risk of T2DM, out of which KLF14, DUSP9, JAZF1, IRS1, and KCNQ1 have strong associations. KLF14 rs972283 has been studied and found to have a significant association with the progression and development of T2DM. In this study, two Pakistani-origin populations were targeted, one of which included Pakistan-based residents while the other one included UK-based resident participants. The genotyping of the samples was carried out using the TaqMan and KASPar methods. PLINK v1.07 or STATA IC version 10.1 were used for statistical analysis while for the SNPs analysis, the Hardy–Weinberg equilibrium was used [81]. For further confirmation and authentication of SNPs in South Asians, more studies and meta-analyses are required.

**Figure 3:**
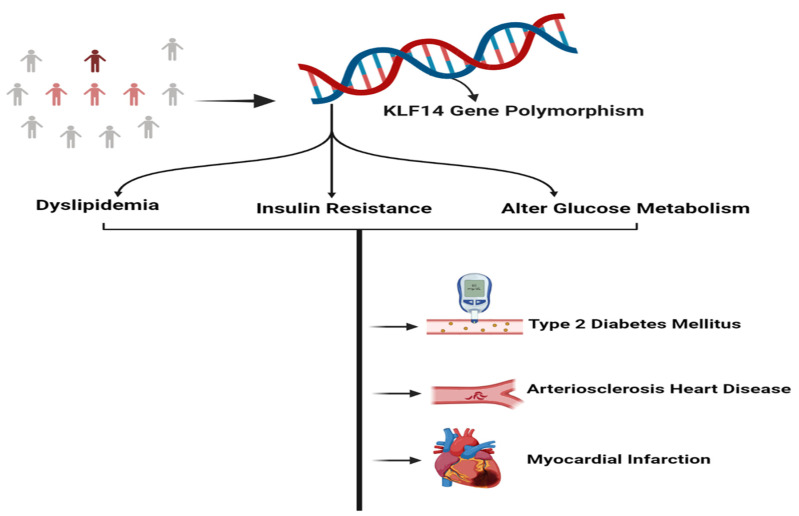
Association of genetic polymorphisms in the KLF14 gene with the pathogenesis of metabolic disorders.

KLF14 has an important role in the metabolism of lipids and gluconeogenesis. There are number of studies that have been conducted on different populations to determine the incidence rate of KLF14 gene polymorphisms and its association with altered lipid levels and the progression of metabolic disorders. In the Han and the Guangxi Mulao populations, an investigational study has been carried out to determine the association between KLF14 rs4731702 SNP and serum lipid levels. A total of 1467 participants were recruited for this study, and the correlation between environmental conditions, serum lipid levels, and genotypes was carried out using multiple regression analysis. The results found that the association of serum lipid levels with KLF14 rs4731702 is inconsistent among these two ethnic populations, which may be due to the ethnic specificity or diverse interactions between the genome and environmental conditions. However, the overall allelic and genomic frequencies of the SNP between the Han and Mulao populations were the same [82]. In the Argentinean population, a study has been conducted to determine the association between KLF14 rs4731702 SNP and the serum lipid profile in diabetic patients. The results revealed that the altered serum lipoprotein profile may be due to the lower activity of KLF14 in diabetic patients. As KLF14 is an insulin-sensitizing transcription factor, the C/C genotype of rs4731702 in diabetic patients shows significant insulin resistance and, in the future, is more prone to develop critical pathologies regarding altered lipoproteins profile such as severe CADs (Figure 3).

In this study, for genotyping analysis, TETRA-ARMS PCR was used, and for statistical analysis of the data, the chi-squared test was used. For the determination of the association between the biochemical parameters and the KLF14 genotype, Fisher’s exact test and t-tests were used [83]. Different experimental studies that determined the KLF14 gene polymorphism in different ethnicities are enlisted in the Table 2.

## 12. Conclusions

KLF14 is a master trans-regulatory gene, which is involved in the regulation of several biological processes and cellular pathways. KLF14 regulates several important mechanisms, including insulin secretion, lipid metabolism, and glucose regulation. KLF14 can be considered as a target for the treatment of cancer as it regulates cell apoptosis, cell proliferation, and differentiation. Moreover, KLF14 has age-related CpG sites, which become methylated with the age. Thus, this gene can be used for the estimation of age in humans. Any alteration or dysregularities in KLF14 induce metabolic disorders such as T2DM and atherosclerotic heart diseases. One of the severe gene irregularities is genetic polymorphism, and a number of studies have been conducted which found that KLF14 SNPs are strongly associated with the development and progression of metabolic disorders. Further investigational studies should be conducted to evaluate the pathological causes of gene polymorphism and its ultimate therapeutic treatment.

## Figures and Tables

**Table 1:** Impact and ultimate consequences of KLF14 genetic variants on Trans-regulatory genes.

Sr.#	Tans- Regulator Gene	Gene Encoded Protein	Gene Expression	KLF14 Interaction	Mutant KLF14 Impact on Gene Expression	Consequences	Ref.
1.	IDE	Zinc metallopeptidase (Insulin degrading enzyme)	IDE degrades peptides, including insulin, glucagon, and amylin. Moreover, it regulates pancreatic β cells functions	KLF14 binds with promoter site of IDE gene	Impaired KLF14 induces irregularities in the normal IDE gene expression	Ultimate outcomes are glucose intolerance, impaired insulin degradation, insulin resistance, and type 2 diabetes mellitus inducement	[10,39]
2.	SLC2A4	GLUT4	Regulates the insulin sensitive facilitative glucose transportation in fats and muscle cells	The promoter region of SLC2A4 gene has binding affinity for KLF14	KLF14 regulates the SLC2A4 normal expression, any genetic variations in KLF14 lead toward compromised SLC2A4 gene expression in glucose regulation	Glucose intolerance which leads to type 2 diabetes mellitus development and obesity	[10,40]
3.	STARD10	StAR-related lipid transfer protein 10	This phosphoinositide-binding protein regulates insulin secretion and synthesis	KLF14 has an affinity to bind with the promoter region of the STARD10 trans-gene	Mutant KLF14 binding on the promoter region of the gene increases the risk of type 2 diabetes mellitus by disrupting the normal gene expression	Glucose intolerance, impaired glucose-induced insulin secretion, and type 2 diabetes mellitus	[10,41]

**Table 2:** Association of KLF14 gene polymorphism among different populations.

Sr. #	Associated SNP	Ethnicity	Allele	Analysis Method	Sample Size	Study Design	Findings	Future Aspects	Ref.
1.	rs972283	Argentina	A/G	Tetra-primer PCR	A total of 50 participants in which 25 were controls and 25 were diseased	Cross-sectional	KLF14 rs972283 (A/G) polymorphism has been found in the type 2 diabetic population of Argentina	Diabetic patients with the KLF14 rs972283 gene polymorphism are more prone to develop cardiac diseases in future	[84]
2.	rs4731702	San Luis, Argentina	C/T	Tetra Primer ARMS-PCR	A total of 60 in which 30 were controls and 30 diseased	Cross-sectional	KLF14 rs4731702 (C/T) polymorphism has been found in the type 2 diabetic population of Argentina	Diabetic patients with theKLF14 rs4731702 gene polymorphism are more prone to develop atherosclerotic and coronary artery diseases in the future	[85]
3.	rs972283	Han Chinese, Henan province	A/G	TaqMan PCR	A total of 1504 in which 768 were controls and 736 diseased	Large case control study	No association between KLF14 rs972283 and type 2 diabetes mellitus has been found	In the Henan province population, KLF14 rs972283 gene polymorphism has not prevailed. Hence, the KLF14 gene has no association with development of cardiac problems among the diabetic population	[78]
4.	rs972283	Globally	A/G	Fixed and random effects meta-analysis	A total of 5 studies with 106,535 controls and 50,552 diseased	Global meta-analysis	KLF14 rs972283 (A/G) polymorphism association with the increased risk of type 2 diabetes has been found throughout the world	Globally, diabetic patients are at high risk of developing cardiac illnesses	[79]
5.	rs4731702	Han and Guangxi Mulao	C/T	RFLP-PCR	A total of 1467 subjects, 740 from the Han and 727 from the Guangxi population	Cross-sectional study	Both of the populations show the same allele frequency pattern for KLF14 rs4731702 but there is a higher allele frequency in females than males	In the Han and Guangxi Mulao populations, females have more KLF14 rs4731702 frequency differences, thus they are more prone to develop cardiac problems and atherosclerosis	[82]
6.	rs1364422	Taiwan	T/C	Affymetrix Axiom genotyping array	A total of 41,526 participants	Genome-wide association study	The number of genes including the KLF14 gene was examined to determine the gene polymorphism frequency and results found that KLF14 rs1364422 has a strong association with the risk of type 2 diabetes especially in females	In females of the Taiwan population, KLF14 rs1364422 gene polymorphism altered the metabolic factors including HDL-C, which can lead to the development of cardiac diseases, especially in diabetic female patients	[86]
7.	rs76603546	Iranian population	C/T	RFLP-PCR	A total of 2064 participants	Case control study	In the Iranian population, KLF14 rs76603546 gene polymorphism has been found	KLF14 rs76603546 has been associated with an increased risk of type 2 diabetes mellitus and altered levels of Hb1AC and BMI	[80]
8.	rs13233731	European population	G/A	Large scale genotyping using metabochip	A total of 149821 participants, in which 56862 were controls and 12171 were diseased	Large scale meta-analysis	In the European population, KLF14 rs13233731 has been found as a risk of type 2 diabetes mellitus	The meta-analysis study has found KLF14 rs13233731 SNP in the European population, which can induce metabolic disorders especially diabetes and cardiac disorders	[87]
